# Supplementary material for: PERK-mediated antioxidant response is key for pathogen persistence in ticks
Source: mSphere. 2023 Sep 21;8(5):e00321-23. doi: 10.1128/msphere.00321-23 (PMC10597351; doi:10.1128/msphere.00321-23)
Supplement: Supplemental figure legends — Legends for Figures S1-S4. [file msphere.00321-23-s0001.docx]

**Supplemental Figure 1 | Sequence alignment of eIF2α from *I. scapularis* and *Homo sapiens*.** Available protein sequences from NCBI were imported into Jalview to visualize sequence alignments. Shaded amino acid residues indicate conservation index and percentage identity between the two proteins. The activity-inducing phosphoserine of eIF2α, Ser51, is indicated with black shading.

**Supplemental Figure 2 | *Ixodes* Nrf2 sequence alignment and structural prediction with AlphaFold.** (**A**) Human and *Ixodes* Nrf2 protein sequences were aligned and visualized with Jalview. The conservation index and percentage identity between the two proteins is indicated by shaded amino acid residues. Amino acids that mediate DNA interactions with promoter ARE regions are indicated with black shading. (**B**) *Ixodes* Nrf2 protein as predicted by AlphaFold. Each residue is color-coded based on the model confidence score, pLDDT. Blue indicates the most confidently predicted regions. Orange to yellow indicates regions of low confidence.

**Supplemental Figure 3 | ISRIB does not potentiate ROS or RNS in tick cells.** ROS (**A**) and RNS (**B**) measurements in ISE6 cells (1.68 x 10^5^). Cells were untreated (-) or pretreated with 1µM ISRIB. Fluorescence was measured at the indicated time points and is presented as RFU, normalized to untreated controls (-).

**Supplemental Figure 4 | Exogenous N-acetyl cysteine reduces the ROS caused by silencing PERK.** ROS measurements in IDE12 cells (1.68 x 10^5^). Cells were treated with silencing RNA targeting *perk* or scrambled controls (scRNA) for 24 hours. Cells were infected with *A. phagocytophilum* (**A**) or *B. burgdorferi* (**B**) alone or in the presence of NAC. Fluorescence was measured at 24 hours post-infection and is presented as RFU, normalized to scrambled RNA controls. Data are representative of 4 biological replicates. Error bars show SEM, *P < 0.05 (Student’s t-test). NAC, N-acetyl cysteine. scRNA, scrambled RNA; siRNA, small interfering RNA.
