# Supplementary figures and images for: PERK-mediated antioxidant response is key for pathogen persistence in ticks
Source: mSphere. 2023 Sep 21;8(5):e00321-23. doi: 10.1128/msphere.00321-23 (PMC10597351; doi:10.1128/msphere.00321-23)

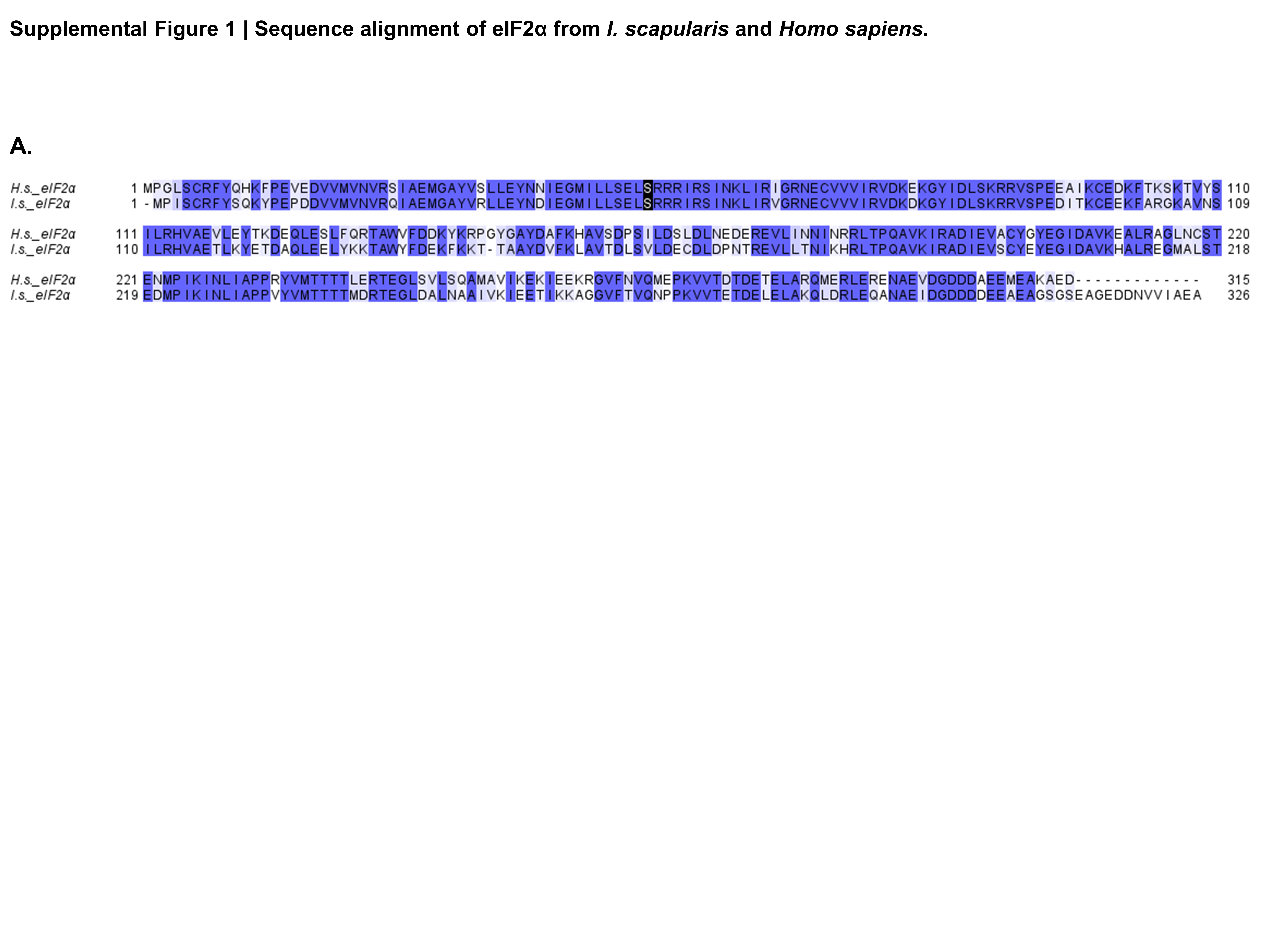

Supplement: Figure S1 — Sequence alignment of eIF2α from I. scapularis and Homo sapiens. [file msphere.00321-23-s0002.tif]

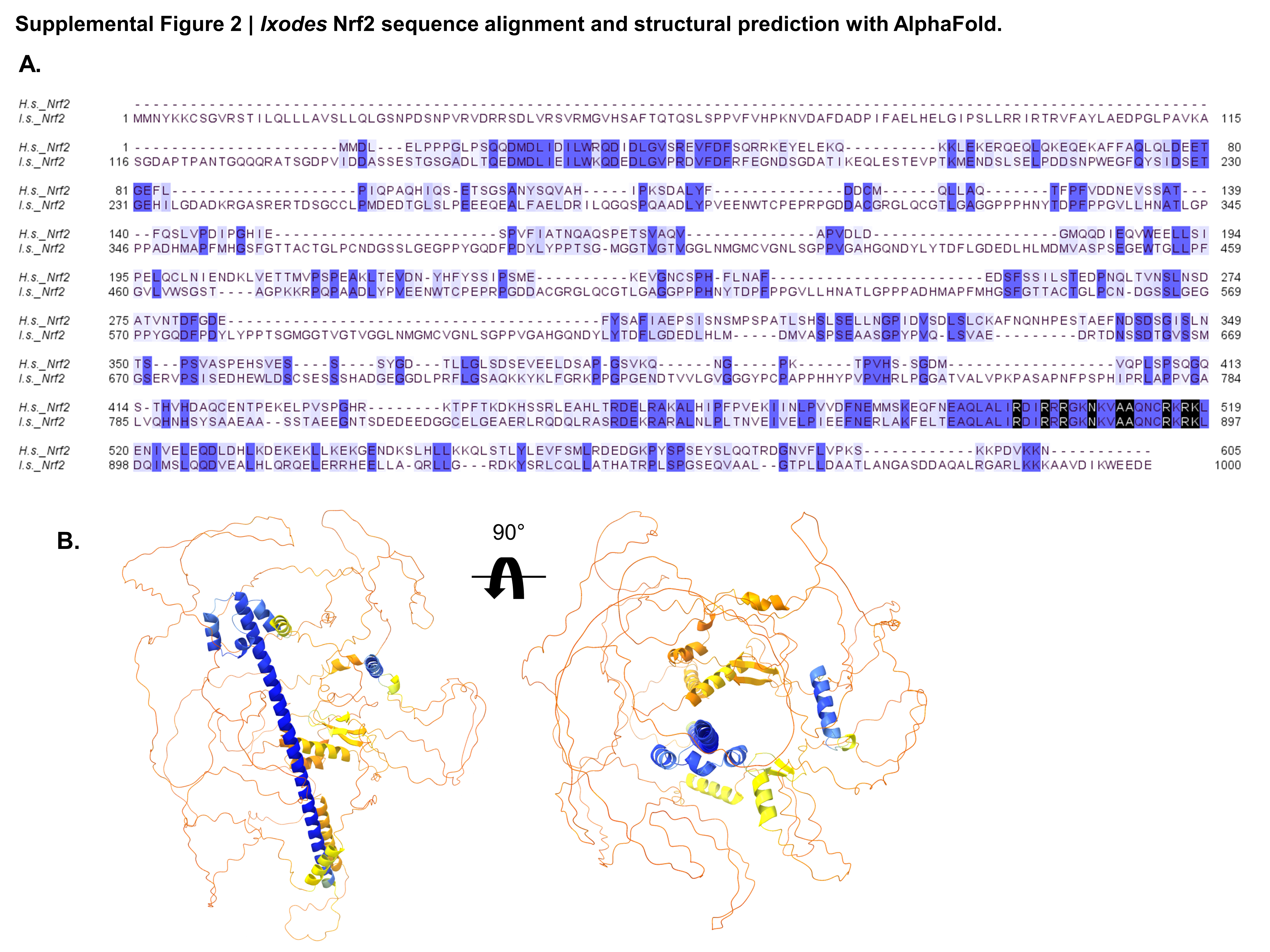

Supplement: Figure S2 — Ixodes Nrf2 sequence alignment and structural prediction with AlphaFold. [file msphere.00321-23-s0003.tif]

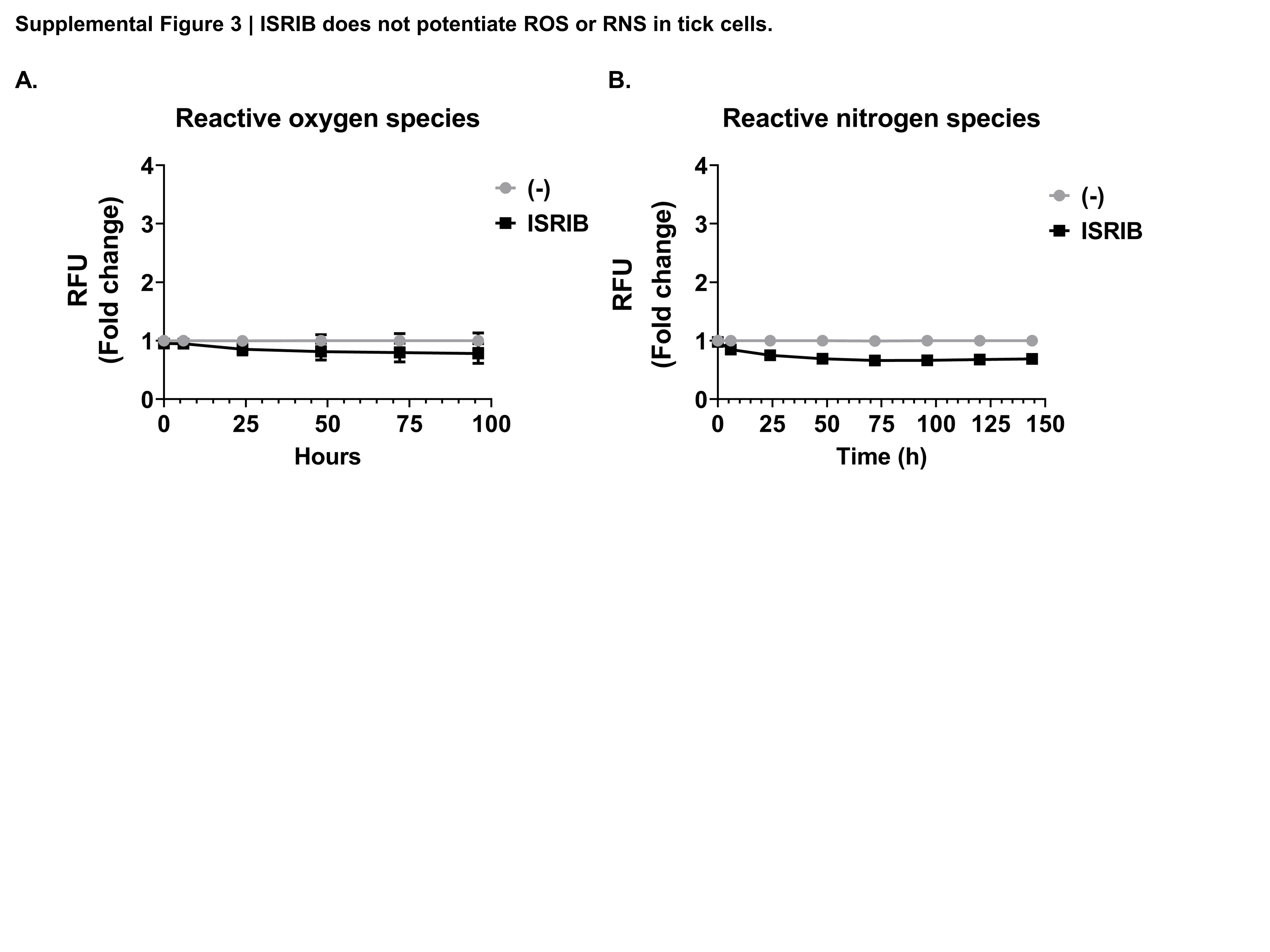

Supplement: Figure S3 — ISRIB does not potentiate ROS or RNS in tick cells. [file msphere.00321-23-s0004.tif]

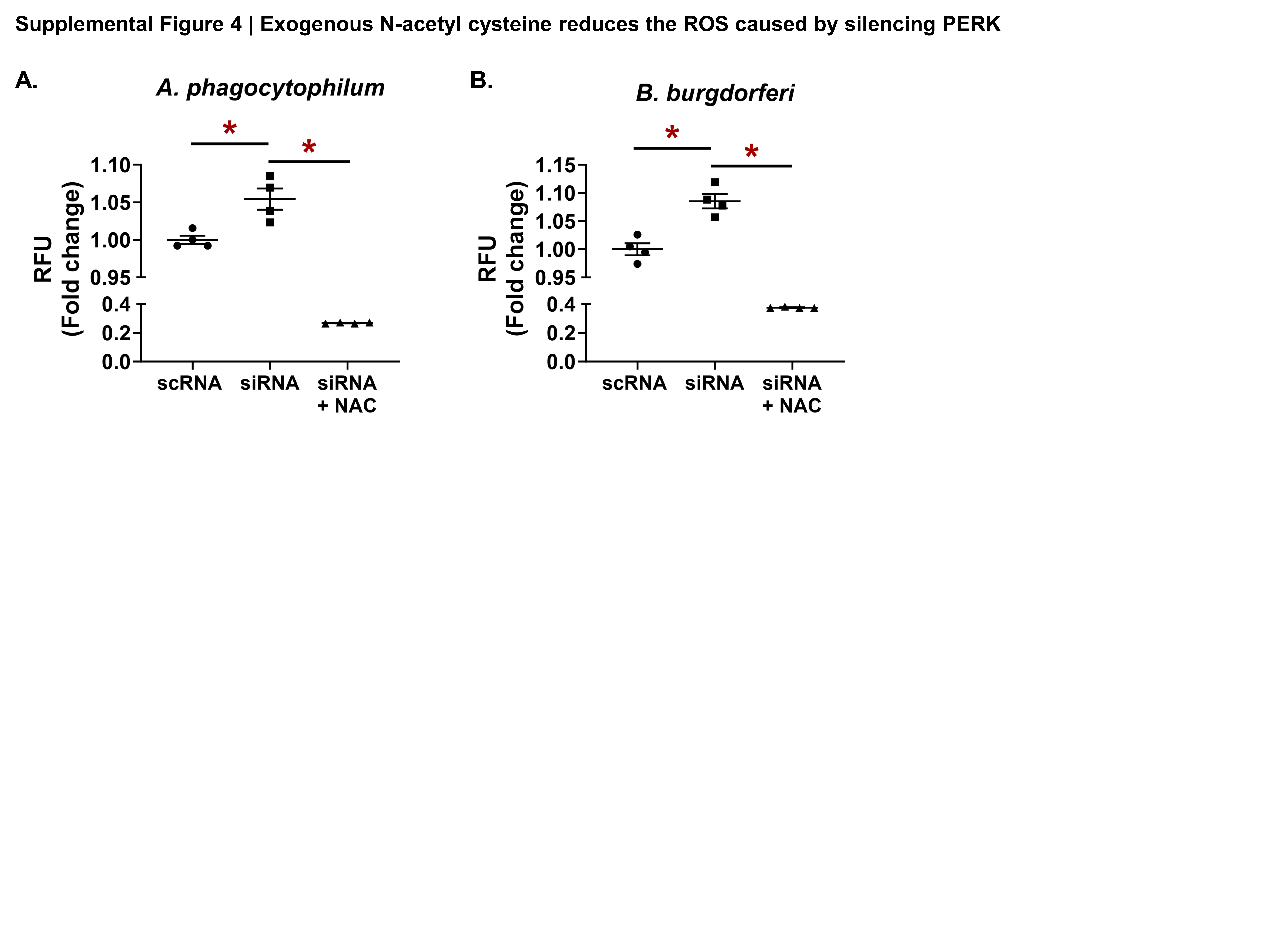

Supplement: Figure S4 — Exogenous N-acetyl cysteine reduces the ROS caused by silencing PERK. [file msphere.00321-23-s0005.tif]
