# Supplementary material for: PERK-mediated antioxidant response is key for pathogen persistence in ticks
Source: mSphere. 2023 Sep 21;8(5):e00321-23. doi: 10.1128/msphere.00321-23 (PMC10597351; doi:10.1128/msphere.00321-23)
Supplement: Table S1 — Oligonucleotide primers used in this study. [file msphere.00321-23-s0006.pdf]

Supplemental Table 1. Oligonucleotide primers used in this study.

Name	Target gene	Primer Sequences
Mus musculus β-Actin (qRT-PCR)	XM_030254057.1	F 5'-ACGCAGAGGGAAATCGTGCGTGAC-3'<br>R 5'-ACGCGGGAGGAAGAGGATGCGGCAGTG-3'
Anaplasma phagocytophilum 16S (qRT-PCR)	NC_007797	F 5'-CCCTAAGGCCTTCCTCACTC-3'<br>R 5'-CAGCCACACTGGAACTGAGA-3'
Anaplasma phagocytophilum 16S_full	NC_007797	F 5'- TCCTGGCTCAGAACGAACG-3'<br>R 5'- GTCACTGACCCAACCTTAAATGG-3'
Borrelia burgdorferi FlaB (qRT-PCR)	MN954474.1	F 5'-TTGCTGATCAAGCTCAATATAACCA-3'<br>R 5'-TTGAGACCCTGAAAGTGATGC-3'
Ixodes scapularis Actin (qRT-PCR)	XM_029977298.1	F 5'-GCCGGGACCTTACAGACTATC-3'<br>R 5'-CACGGACAATTTCACGCTCG-3'
Ixodes scapularis IRE1α (qRT-PCR)	XM_029972190.1	F 5'-GAGAAGGCCATCTTCGTCGG-3'<br>R 5'-GAGTAGCCTGGGCAGCATAG-3'
Ixodes scapularis TRAF2 (qRT-PCR)	XM_029977983.1	F 5'-CCGCGAAAAGAACAGCTTAC-3'<br>R 5'-TACCACGTTGGACTCCTTCC-3'
Ixodes scapularis BiP (qRT-PCR)	XM_002433611.2	F 5'-ATCGTGTGGTGTCTAGCGG-3'<br>R 5'-CGATACCGATGACTGTGCCG-3'
Ixodes scapularis PERK (qRT-PCR)	XM_029994352.4	F 5'-ATCCACTCCTGTACTTGGGC-3'<br>R 5'-TTCACCCGATTCTGAAGGCT-3'
Ixodes scapularis HRI (qRT-PCR)	XM_029992945.4	F 5'-TATTCCGAAGCTGTCTCCGC-3'<br>R 5'-GTCAGCAGGTTGTAGCCCTA-3'
Ixodes scapularis GCN2 (qRT-PCR)	XM_029969993.4	F 5'-GCCCCAAGAAAGCATGACTC-3'<br>R 5'-TGCTTCGTTGGGATACTGGT-3'
Ixodes scapularis eIF2α (qRT-PCR)	XM_040212362.2	F 5'-ATCCGGTCCATCAACAAGCT-3'<br>R 5'-TCCTTGTCCACCCTGATGAC-3'
Ixodes scapularis ATF4 (qRT-PCR)	XM_029967345.4	F 5'- AGGTTGTCTACTGCCCGTAC-3'<br>R 5'- TCCCAGTCGACTTCCATGTC-3'
Borrelia burgdorferi cp9 (PCR)	BBC10	F 5'-GAACTATTTATAATAAAAAGGAGAGC-3'<br>R 5'-ATCTTCTTCAAGATATTTTATTATAC-3'
Borrelia burgdorferi cp26 (PCR)	BBB19	F 5'-AATAATTCAGGGAAAGATGGG-3'<br>R 5'-AGGTTTTTTTGGACTTTCTGCC-3'
Borrelia burgdorferi lp17 (PCR)	BBD10	F 5'-CAAACTTATCAAATAGCTTATC-3'<br>R 5'-ACTGCCACCAAGTAATTTAAC-3'
Borrelia burgdorferi lp25 (PCR)	BBE16	F 5'-ATGGGTAAAATATTATTTTTTGGG-3'<br>R 5'-AAGATTGTATTTTGGCAAAAAATTTTC-3'
Borrelia burgdorferi lp28-1 (PCR)	BBF20	F 5'-ATGAACAAAAAAATTTTCTATTTC-3'<br>R 5'-GTTGCTTTTGCAATATGAATAGG-3'
Borrelia burgdorferi lp28-2 (PCR)	BBG02	F 5'-TCCCTAGTTCTAGTATCTACTAGACCG-3'<br>R 5'-TTTTTTTTGTATGCCAATTGTATAATG-3'
Borrelia burgdorferi lp28-3 (PCR)	BBH06	F 5'-GATGTTAGTAGATTAAATCAG-3'<br>R 5'-TAATAAAGTTTGCTTAATAGC-3'
Borrelia burgdorferi lp28-4 (PCR)	BBI16	F 5'-CAGGCCGGATTTTAATATCGA-3'<br>R 5'-GTTTATATTTTGACACTATAAG-3'
Borrelia burgdorferi lp36 (PCR)	BBK19	F 5'-AAGTTTATGTTTATTATTGC-3'<br>R 5'-ATTGTTAGGTTTTTCTTTTCC-3'
Borrelia burgdorferi lp38 (PCR)	BBJ34	F 5'-AAATTCTATGGAAGTGATG-3'<br>R 5'-TTTCTATTTATTTTTAGGC-3'

Borrelia burgdorferi Ip54 (PCR)	BBA16	F 5'-GCACAAAAAGGTGCTGAG-3'<br>R 5'-TTTTAAAGCGTTTTTAAGC-3'
Borrelia burgdorferi Ip56 (PCR)	BBQ56	F 5'-AAGATTGATGCAACTGGTAAAG-3'<br>R 5'-CTGACTGTAACTGATGTATCC-3'
Ixodes scapularis eIF2α _siRNA_438	XM_040212362.2	F 5'-AACCGGAAGACATCACGAAATCCTGTCTC-3'<br>R 5'-AAATTTCGTGATGTCTTCCGGCCTGTCTC-3'
Ixodes scapularis eIF2α_ scRNA	N/A	F 5'-AAGCATAGCGGAACCTAACAACCTGTCTC-3'<br>R 5'-AATTGTTAGGTTCCGCTATGCCCTGTCTC-3'
Ixodes scapularis ATF4_siRNA_863	XM_029967345.4	F 5'-AAGCAGAGTCCTTTCCGGAAACCTGTCTC-3'<br>R 5'-AATTTCCGGAAAGGACTCTGCCCTGTCTC-3'
Ixodes scapularis ATF4_ scRNA	N/A	F 5'-AAGGCACAGCTTCGCAGTAATCCTGTCTC-3'<br>R 5'-AAATTACTGCGAAGCTGTGCCCCTGTCTC-3'
Ixodes scapularis PERK_siRNA_1064	XM_029994352.4	F 5'-AAGCATAGAATGGAAGCCCTACCTGTCTC-3'<br>R 5'-AATAGGGCTTCCATTCTATGCCCTGTCTC-3'
Ixodes scapularis PERK_scRNA	N/A	F 5'-AAGGTAACAAACCGGAGTCATCCTGTCTC-3'<br>R 5'-AAATGACTCCGGTTTGTTACCCCTGTCTC-3'
Ixodes scapularis HRI_siRNA_231	XM_029992945.4	F 5'-AAGGATCATACTCCTGATCTTCCTGTCTC-3'<br>R 5'-AAAAGATCAGGAGTATGATCCCCTGTCTC-3'
Ixodes scapularis HRI_scRNA	N/A	F 5'-AAACTATTCGTCTACTCGGTACCTGTCTC-3'<br>R 5'-AATACCGAGTAGACGAATAGTCCTGTCTC-3'
Ixodes scapularis GCN2_siRNA_372	XM_029969993.4	F 5'-AACCAACACAATACACCTCAACCTGTCTC-3'<br>R 5'-AATTGAGGTGTATTGTGTTGGCCTGTCTC-3'
Ixodes scapularis GCN2_scRNA	N/A	F 5'-AAACCAACATACCATAACACCCCTGTCTC-3'<br>R 5'-AAGGTGTTATGGTATGTTGGTCCTGTCTC-3'
Ixodes scapularis Nrf2_siRNA_1058	XM_042293400.1	F 5'-AACCTTCATGCATGGATCCTTCCTGTCTC-3'<br>R 5'-AAAAGGATCCATGCATGAAGGCCTGTCTC-3'
Ixodes scapularis Nrf2_scRNA	N/A	F 5'-AAGCTTACCGAGTCTCCTATTCCTGTCTC-3'<br>R 5'-AAAATAGGAGACTCGGTAAGCCCTGTCTC-3'
Ixodes scapularis Nrf2 (qRT-PCR)	XM_042293400.1	F 5'-GTCTTCGACTTCCGGTTTGA-3'<br>R 5'-GTAGGCACTTCGGTGCTCTC-3'
